# Risk of canine and human exposure to *Dirofilaria immitis* infected mosquitoes in endemic areas of Italy

**DOI:** 10.1186/1756-3305-6-60

**Published:** 2013-03-07

**Authors:** Gioia Capelli, Antonio Frangipane di Regalbono, Giulia Simonato, Rudi Cassini, Stefania Cazzin, Gabriella Cancrini, Domenico Otranto, Mario Pietrobelli

**Affiliations:** 1 Istituto Zooprofilattico Sperimentale delle Venezie, Legnaro, Padua, Italy; 2 Department of Animal Medicine, Production and Health, University of Padua, Legnaro, Padua, Italy; 3 Department of Comparative Biomedicine and Food Science, University of Padua, Legnaro, Padua, Italy; 4 Department of Public Health and Infectious Diseases-Parasitology Section, University of Rome “Sapienza”, Rome, Italy; 5 Department of Veterinary Medicine, University of Bari, Bari, Valenzano, Italy

**Keywords:** *Dirofilaria immitis*, Mosquito vectors, Dogs, Humans, Risk of exposure

## Abstract

**Background:**

The occurrence of infections by *Dirofilaria immitis* in canine and human populations depends on several factors linked to both the definitive and intermediate hosts. Little data are available on the risk of human and dog exposure to *D. immitis* in endemic areas. Data collected on dog- and human-bait traps in endemic areas of north-eastern Italy were used to estimate the likelihood of a receptive host coming into contact with an infected vector.

**Methods:**

From 1997 to 1999, mosquitoes were collected from three sampling sites of north-eastern Italy on *D. immitis* microfilaraemic dogs and on human baits. The bite/night/host rates were determined based on the number of feeding and probing mosquitoes on dogs and humans, respectively. The survival/mortality rates of different species of mosquitoes following the blood meal, and the rate of natural *Dirofilaria* infection in unfed specimens were estimated. The risk of exposure of dogs and humans to infected mosquito species was determined by combining the bite/host/night and the mosquito infection rates.

**Results:**

A total of 1,165 mosquitoes were collected on human (n = 815) and dog (n = 350) baits with varying species composition (i.e., *Culex pipiens*, 87.3% and *Ochlerotatus caspius*, 11.6%). Overall, dogs were more attractive to *Cx pipiens* than humans (feeding rate 70.2% *vs* probing rate 25.9%). The highest bite/night/host rate was 84.0 for dogs and 26.5 for humans. *Cx pipiens* displayed a mortality rate of 76.3% within 13 days and *Oc. caspius* of 100% within two days following the infective blood meal. In addition, *D. immitis* DNA was detected in unfed *Cx pipiens* (infection rate of 0.26%-2.07%). The infection rate adjusted for mosquito mortality was 0.38%. Based on data collected, the contact between an infected mosquito and a host can occur as often as every four nights for *D. immitis* infected-mosquitoes in dogs and within two weeks for humans.

**Conclusions:**

*Cx pipiens* was confirmed as the most efficient natural vector of *D. immitis* in the studied area. In endemic areas, the risk of transmission can be very high for dogs and relevant for humans. Despite the increased awareness of veterinarians and owners on canine dirofilarioses, dogs from rural areas still maintain the natural life cycle of *Dirofilaria* spp., therefore acting as a source of infection to humans through vector bites.

## Background

*Dirofilaria immitis* (Filarioidea, Onchocercidae) is responsible for cardiopulmonary dirofilariosis. This filarioid is transmitted by many species of mosquito vectors (genus *Culex, Aede*s, *Ochlerotatus* and *Anopheles*), in which it develops into the infective third stage within different timeframes, which depend upon several factors [1-4]. Among vector-borne helminths, *Dirofilaria* spp. have been recognised as emerging zoonotic agents, currently spreading throughout Europe [5,6]. The risk of canine and human infection by *D. immitis* is linked to a combination of several factors related with both the definitive and the intermediate hosts. For example, the rates of infection in the intermediate host depend on vector densities, host-seeking activity/feeding preference, and vector competence [4,7,8]. Mosquito species of the genus *Culex* and *Ochlerotatus* have been reported as major vectors of *Dirofilaria* in Italy and other European countries [3,9,10]. Current data on the vectors of *Dirofilaria* spp. derives from laboratory experiments, occasional findings in naturally infested insects, or from fieldworks using dogs and/or humans-bait traps [11]. In these studies, the rate of mosquito infection has been estimated by insect dissection or biomolecular methods. Nevertheless, the risk of dogs and humans to be exposed to *D. immitis* infected vectors in endemic areas, has never been investigated.

In the present study, data on risk for dog and human exposure to *D. immitis* infected mosquitoes in endemic areas of Italy have been examined and discussed.

## Methods

### Sampling area and mosquito collections

From 1997 to 1999, nocturnal mosquito collections were carried out in lowland areas of north-eastern Italy, endemic for dirofilariosis [5,6,10]. Dog- and man-attracted mosquitoes were collected in three peri-urban sites, i.e. Rodeano (site A; province of Udine, Friuli Venezia Giulia region; 46°06'43"N −13°00'13"E, 130 m above sea level [a.s.l.]), Piove di Sacco (site B; province of Padua; Veneto region; 45°17'49"N–12°02'06"E, 5 m a.s.l.), and Sarzano (site C; province of Rovigo; Veneto region; 45°04'50"N–11°49'38"E, 5 m a.s.l.). The mosquito collection started in each site when a minimum temperature of >15°C was recorded, and terminated at the end of September of each year. Microfilaraemic dogs naturally infected by *D. immitis* (i.e., a 7 year old male pure-breed dog (Bobtail), in site A; a 5 year old male cross-breed dog (medium size), in site B; and a 6 year old male cross-breed dog (small size), in site C), were used as bait in each sampling site. Average values of microfilaraemia, expressed as number of microfilariae per milliliter (mf/ml), were calculated by ten counts serially performed on 10 μl of blood samples collected from each dog at the beginning and at the end of the study.

**Figure 1:**
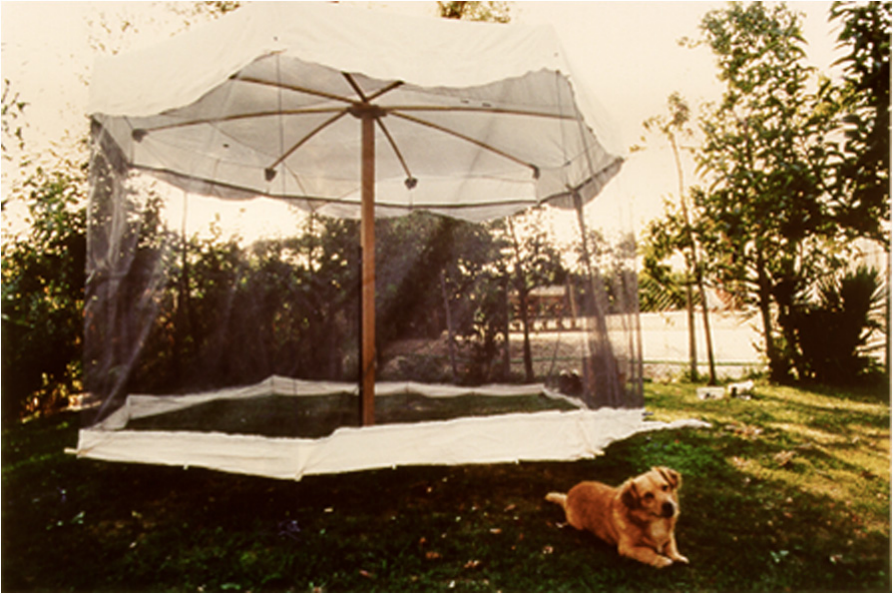
Dog bait trap (site C).

Mosquitoes were also collected while landing on two persons. The same persons were employed in site B and C, whereas one of the two persons was replaced in site A. One dog- and one man-bait trap were employed simultaneously in the three sites for 17 sampling nights, i.e., for six nights in site A (August 5-September 15, 1997), six nights in site B (July 29-September 23, 1998), and five nights in site C (June 23-September 15, 1999). The traps consisted of a cylindrical structure of wood (2.3 m of diameter × 2.0 m in height for dog and 2.0 m × 1.5 m for humans) covered by a net, five cm above the ground to allow mosquitoes to enter the trap [12,13] (Figure 1). The risk of mosquitoes escaping from the trap was considered negligible, due to both the insect host-seeking behaviour (in which mosquitoes rarely fly downwards when leaving an enclosed space), and to the fact that engorged females do not move after the blood meal, resting on the net [14]. In each site, the human and dog baits were located at a distance of at least ten meters to avoid interference between mosquito attractants.

Mosquito collections were performed using a paper cup aspirator [15] from 8:00 p.m. until 6:00 a.m. In dog-bait traps engorged insects resting on the net were collected every two hours. Unfed mosquitoes were left in the traps until the last sampling (6:00 a.m). Mosquitoes were collected by using the above procedures in the human-bait traps, with two persons acting as bait and collectors, simultaneously. Only mosquitoes attempting to probe on humans were collected.

### Laboratory procedures

The mosquitoes were identified according to morphological keys [16]. The unfed mosquitoes collected in dog-bait traps and all the mosquitoes collected in human-bait traps (probing and resting on the net) were pooled (minimum 1 - maximum 12 specimens) according to species, date and site of collection.

The numbers of human-probing mosquitoes and of dog-fed specimens were used to evaluate the bite/night/host rates. Alive dog-fed insects were kept under standard insectary conditions (25–27°C, ∼90% relative humidity) for 13 days, and observed daily in order to estimate the mortality rate after the microfilaraemic blood meal [17].

The DNA was extracted separately from pools of the insect abdomens and thorax-heads, respectively, in order to detect potentially infective specimens [18].

The pooled samples were analysed by PCR amplification with *D. immitis* and *D. repens* specific pair of primers (R1-R2, and I1-I2, respectively) as previously described [19]. All sequences generated were compared to sequences available in GenBank using standard BLAST searches.

### Ethical statement

The procedures for sampling mosquitoes attracted to humans and dogs followed a standard protocol [12]. At the time of the study, in absence of any Ethical for Animal Experimentation Committee at the University of Padua, the study was performed according to the legislative decree n. 116 (27 January 1992), implementing the Council directive n. 86/609/EEC on the protection of animals used for experimental purposes. All humans involved in the field study were staff employed by the Faculty of Veterinary Medicine of Padua and provided their informed consent to all components of the study. Since in human bait traps two persons collected mosquitoes while probing and not following feeding, the infection risk with any pathogen was considered negligible. The dogs used in the study were naturally infected by *D. immitis* and were usually kept outdoors during the night. Dogs were neither anesthetized nor forced under the traps, which were placed at their usual sleeping site. The dog owners declared their unwillingness to treat them against dirofilariosis. However, a free treatment was offered to dogs at the end of the study.

### Statistical analyses

The differences among the proportions of mosquitoes that fed on dogs or landed on humans were tested by the chi-square test or the Fisher exact test when appropriate [20]. The mean numbers of different mosquito species captured on dogs and humans were compared by ANOVA, after log_e_ (× + 1) transformation of the data (software SPSS, version 13.0 for windows).

The rate of infection in mosquitoes was adjusted for pooled samples, calculating the Estimated Rate of Infection (ERI) using the following formula [21]:

ERI = 1-(1-n/N)^1/k^, where n is the number of positive pools, N the number of examined pools, and k the average number of specimens in each pool [22]. Before calculating ERI for *D. immitis* the positive pools detected from the pooled mosquito abdomens (infected and not still infective) was adjusted with the mean mortality rate found in reared *Cx pipiens*.

The exposure risk, i.e., the risk of contact with an infected mosquito species, was calculated by combining the bite/dog/night and the bite/human/night rates at each sampling with the ERI, as follows:

days to contact a *Dirofilaria* spp. infected mosquito = 100/(bite/host/night)/ERI

The ERI calculated in this study was used for site C, while the ERI estimated in a separate study performed in the same area in 2010 [11] was used for site B. No data on mosquito ERI is available for site A.

The risk of exposure was expressed as “risk in days” (i.e., the minimum number of nights of exposure necessary to come in contact with an infected mosquito under the specific epidemiological conditions of each site at each data time/point). Therefore, the shorter the time, the higher the risk of contact.

## Results

The dogs trapped in site A, B and C displayed a microfilaraemia of 30–35,000; 100–110,000 and 110–125,000 mf/ml, at the beginning and at the end of the study, respectively.

Of the 1,165 mosquitoes captured on host-baits (i.e., 350 on dogs and 815 on humans) the most represented species was *Culex pipiens* (n = 1017; 87.3%), followed by *Ochlerotatus caspius* (n = 135; 11.6%), *Cx modestus* (n = 5; 0.4%), *Coquillettidia richiardii* (n =4; 0.3%) and *Aedes vexans* [*Aedimorphus vexans*] (n = 4; 0.3%). The latter two species were captured on humans only (Table 1).

**Table 1:** Total number and species of mosquitoes collected using dog- and human- bait traps and number and proportion (%) of fed (dog) or landed (man) mosquitoes

	**Dog (n = 3)**			**Man (n = 6)**^**a**^		
	**Total n. mosquitoes**	**Fed**	**%**	**Total n. mosquitoes**	**Landed**	**%**
*Culex pipiens*	329	231	70.2*	688	178	25.9*
*Ochlerotatus caspius*	20	18	90.0	115	92	80.0
*Coquillettidia richiardii*	0	0	-	4	4	100.0
*Culex modestus*	1	1	100.0	4	4	100.0
*Aedes vexans*	0	0	-	4	3	75.0
total	350	254	72.6*	815	281	34.5*

^a^ Two persons per trap.

Statistically significant differences are marked (*) (p < 0.01).

Although the mean number of mosquitoes per dog/human which entered the traps did not differ significantly, the rate of *Cx pipiens* that fed on dogs was more than double that of mosquitoes probing on humans (i.e., 70.2% *vs* 25.9%; p < 0.01) (Table 1). The same tendency (p = 0.053) was observed for *Oc. caspius* (i.e., 90.0% *vs* 68.9%) in site C (Table 2).

**Table 2:** *Culex pipiens *and *Ochlerotatus caspius *collected using dog- and human- bait traps and number and proportion (%) of fed (dog) or landed (man) mosquitoes according to site and night of collection (*)

		***Culex pipiens pipiens***				***Ochlerotatus caspius***			
**Site**	**Date**	**Dog**		**Man**^**§**^		**Dog**		**Man**^**§**^	
	**dd/mm/yy**	**fed/tot**	**%**	**landed/tot**	**%**	**fed/tot**	**%**	**landed/tot**	**%**
Site A	05/08/97	2/12	16.7	0/11.5	0.0	0	-	0	-
(Udine)	13/08/97	10/15	66.7 ^a^	26.5/45.5	58.2 ^a^	0	-	0	-
	20/08/97	1/9	11.1	3.5/8	43.8	0	-	0	-
	17/09/97	3/6	50.0	1.5/3	50.0	0	-	0	-
	22/09/97	1/8	12.5	4/10	40.0	0	-	0	-
	25/09/97	5/7	71.4 ^b^	2/7.5	26.7 ^b^	0	-	0	-
	total	22/57	38.6 ^A^	38/85.5	44.4 ^CD^	0	-	0	-
Site B	29/07/98	84/84	100.0^c^	9.5/68.5	13.9^c^	0	-	2/2	100.0
(Padova)	10/08/98	64/67	95.5^d^	2/4.5	44.4^d^	0	-	3.5/3.5	100.0
	20/08/98	7/7	100.0	1/1	100.0	0	-	0/0.5	0.0
	26/08/98	3/3	100.0	0/0	0.0	0	-	0	-
	23/09/98	0/1	0.0	0/0.5	0.0	0	-	0	-
	total	158/162	97.5^eAB^	12.5/74.5	16.8 ^eC^	0	-	5.5/6	91.7
Site C	23/06/99	20/36	55.6^f^	4.5/75.5	6.0^f^	6/7	85.7	0.5/2.5	20.0
(Rovigo)	29/06/99	6/8	75.0	10.5/24.5	42.9	0	-	4.5/5.5	81.8
	14/07/99	2/35	5.7	1/7.5	13.3	6/6	100.0	10/17	58.8
	27/07/99	16/19	84.2^g^	21.5/69	31.2^g^	6/7	85.7	17.5/21.5	81.4
	19/08/99	7/10	70.0	1.5/7.5	20.0	0	-	3/5	60.0
	15/09/99	0/2	0.0	0/0	-	0	-	0	-
	total	51/110	46.4 ^iB^	39/184	21.2 ^iD^	18/20	90	35.5/51.5	68.9

(*) equal letters correspond to significant difference, p < 0.01 (uppercase = comparison within the same host species at the three sites, vertical; lowercase = comparison among dog/humans at the same site, horizontal).

(^§^) landed and total number of mosquitoes collected per person.

The attractiveness of *Cx pipiens* to dogs and humans varied significantly within the same host species (Table 2). Specifically, the feeding rate of mosquitoes feeding on dogs at site B was higher than that recorded on the other two dogs (i.e., 97.5% *vs* 38.6 at site A and 46.4% at site C). In addition, the two persons at site A were more attractive (landing rate of 44.4%) than the ones at sites B and C (i.e., landing rate of 16.8% and 21.2%, respectively) (Table 2).

The overall mortality rate of fed *Cx pipiens* 13 days following collection was 76.3%. In particular, of the 148 mosquitoes, 95 (64.2%) died spontaneously within one week (Table 3). Mortality rates varied among sites with the highest rate (i.e., 91.3%) recorded in mosquitoes which fed on the dog from site C (i.e., the animal harbouring the largest number of microfilariae) (Table 3). However, a variation of mf density throughout the study period cannot be excluded.

**Table 3:** **Mortality in laboratory reared *Culex pipiens *(n = 148) within 13 days following the blood meal, according to microfilaraemic dogs and site of sampling (mf = microfilariae)**

**Site**	**mf/ml**^**a **^**(x1000)**	**Days of mosquito breeding**														**Tot n. dead**	**Overall mortality rate**
		**0**	**1**	**2**	**3**	**4**	**5**	**6**	**7**	**8**	**9**	**10**	**11**	**12**	**13**		
Site A	30-35	18	4	6		2				1			1			14	77.8%
Site B	100-110	84	3	6	7	6	10	5	5	5	1	2	2	2	3	57	67.8%
Site C	110-125	46		27	6	4	1	2	1						1	42	91.3%
Tot		148	7	39	13	12	11	7	6	6	1	2	3	2	4	113	76.3%

^a^ Microfilaraemic values detected at the beginning and at the end of each mosquito collection.

*Oc. caspius* specimens, collected only at site C, died within two days (data not shown).

Out of the 915 unfed mosquitoes processed by PCR, eight pools of *Cx pipiens* from site C were positive for *D. immitis*, one in thorax/head pools and seven in abdomen-pools, corresponding to an estimated rate of infection (ERI) of 0.26% (95% CI 0.01-1.2) and of 2.07% (95% CI 1.03-4.11), respectively. After adjustment for mortality, the overall ERI for site C was 0.38%; this value was used to estimate the risk of exposure to a *D. immitis* infective *Cx pipiens* at the same site. Conversely, the risk of exposure to *D. immitis* at site B was calculated using the ERI recorded in a study performed 10 years later in the same area [11]. For site A no data on mosquito *Dirofilaria* spp. rate of infection is available.

Based on this data, and according to biting rates and ERI recorded at different sites (Table 4) the risk for dogs and humans to be exposed to *D. immitis* infected *Cx pipiens* could range from a minimum of four to a maximum of 300 days and a minimum of 12 to a maximum of 901 days, respectively (Table 4).

**Table 4:** **– Potential risk of exposure (days) to *Dirofilaria immitis *infected *Culex pipiens *based on fed (dog) or landed (man) mosquitoes per night, according to date and site**

**Sites**	**Date**	**Bites/Night/Host**		**ERI **^(*)^	**Days **^**(§)**^	
	**dd/mm/yy**	**Dog**	**Man**	**%**	**Dog**	**Man**
Site B	29/07/98	84	9.5	0.278	4	38
Padova	10/08/98	64	2	0.111	14	450
	20/08/98	7	1	0.111	129	901
	26/08/98	3	0	0.111	300	-
	23/09/98	0	0	0.421	-	-
Site C	23/06/99	20	4.5	0.38	13	58
Rovigo	29/06/99	6	10.5	0.38	44	25
	14/07/99	2	1	0.38	132	263
	27/07/99	16	21.5	0.38	16	12
	19/08/99	7	1.5	0.38	38	175
	15/09/99	0	0	0.38	-	-

^(*)^ ERI = estimated rate of infection. For site C, ERI was calculated in this study and adjusted for *Cx pipiens* mean mortality; for site B, ERI is from [11].

^(§)^ minimum number of days to come in contact with a *D. immitis* infected mosquito.

## Discussion

The results of this study contribute to current understanding of the epidemiology of *Dirofilaria* spp. in an endemic area of north-eastern Italy. Based on the dog/human attractiveness, on the survival after feeding on dogs with high microfilariae concentrations and on the overall rate of positive mosquitoes, *Cx pipiens* was the most efficient natural vector of *D. immitis* in the studied area, where highly microfilaraemic dogs are expected [7,23]. Although the small number of *Oc. caspius* collected does not allow any definitive conclusion to be drawn, the high mortality rate of this species following a blood meal on a highly microfilaraemic dog indicates that *Oc. caspius* might be an effective vector in non-endemic areas. Indeed, the survival of different vectors was linked to *Dirofilaria* microfilariae concentration in the mosquito species [24].

The results also indicate that in north-eastern Italy, where *Cx pipiens* is most prevalent [25], the risk of exposure to *Dirofilaria* spp. infected vectors can be very high for dogs and relevant for humans.

Indeed, based on our calculations, during the seasons characterized by higher biting pressure, the contact between an infected mosquito and a host may occur as often as every four nights for *D. immitis* in dogs, whereas the possibility for a human to come into contact with an infected mosquito occurs within two weeks of exposure. The higher risk of *Dirofilaria* spp. transmission was recorded in late July and August, in accordance with previous reports [23].

In contrast, in a study in which more than 40,000 culicids collected from May to October 2010 in the areas of site B and C were screened for *D. immitis* and *D. repens*[11], the rates of *Cx pipiens* infection did not vary significantly through the season, indicating that over spring a certain number of dogs may act as a source of infection to suitable vectors. This finding indicates that the mosquito abundance is one of the key factors in the epidemiology of dirofilariosis. Interestingly, the rate of *Cx pipiens* infection with *D. immitis* estimated in this study in 1999 (0.26%-0.38%, site C) was very similar to that calculated more than ten years later (0.21-0.33%) [11]. This finding shows that, in spite of the availability of several chemoprophylactic treatments used for the prevention of canine dirofilarioses in endemic areas, prevalence of microfilaraemic dogs has not decreased significantly in rural areas.

However, since the most conservative approach was used to infer the risk of host exposure to *D. immitis* (i.e. assuming that mosquitoes harboring microfilariae in the abdomen would have suffered the same rate of mortality of *Cx pipiens* fed on high microfilaraemic dogs, 76.3%), is likely that in certain sites, the risk of exposure of dogs and humans is higher than indicated.

In addition, due to the introduction of *Aedes albopictus* [*Stegomya albopicta*] in Italy as well in other European countries [26] the risk of *Dirofilaria* transmission is higher throughout the day time, as a consequence of the fact that this species is diurnal and it acts as natural vector of both filariae in Italy [9,27]. Consequently, categories of dogs and humans previously considered not at risk for dirofilariosis (e.g., animals kept indoors at night or children playing in private and public gardens), should be included in the population at risk of exposure.

Without any doubts, host attractiveness plays a key role in the determination of the population at risk of exposure. Indeed, mosquito host preferences have been well documented, both at species and at individual level [28]. Accordingly, in the present investigation, *Cx pipiens* fed preferentially on dogs (70%) than on humans (26%), and was differently attracted by the three dogs used in the study, independently from their sizes. Besides the individual host attractiveness to mosquitoes, the microfilaraemic status of the dogs may enhance the host-preference of *Cx pipiens*. Accordingly, *Cx pipiens* displayed a higher feeding rate on a microfilaraemic dog, compared to a dog under preventative treatment (i.e., 47% *vs* 6.7-12.9%) [13]. Similarly, the mosquito host attractiveness is also enhanced in human patients infected by *Plasmodium* spp. [29,30] indicating that alterations of the physiological status (disease conditions) and consequently of physiological parameters, may ultimately change the animal cue attractants (fever, sweat and breathing rhythm and odour) to competent vectors. Although these factors might have an impact on the spread of the infection, they remain purely speculative for dirofilarioses.

## Conclusions

The quantification of the most important entomological parameters (i.e., mosquito host preference, biting rate, mortality induced by the pathogen, rate of infection), which affect the risk of transmission of a vector-borne disease to a susceptible host population, is pivotal for the estimation of the dog/human exposure to infected bites.

Data herein presented on the *Dirofilaria* spp. transmission from arthropod to vertebrate host, and vice versa, are of importance for setting prediction models of dirofilariosis in animals and human beings in a given geographical area. The similarity in the mosquito infection rates recorded over a period of ten years suggests that, in rural areas, dogs maintain the natural cycle of *Dirofilaria* spp., which enhances the risk of transmission to humans. Therefore, strategies to minimize the contact between animal/humans and vectors are strongly recommended.

## Competing interests

Authors declare that they have not competing interests.

## Authors’ contributions

AFR and MP conceived the study and performed the field collections, AFR, GCap and DO wrote the manuscript, GCan performed the biomolecular analyses, RC, GS and SC participated to the field and laboratory analyses, GCap performed statistical analyses, all the Authors read and approved the final version of the manuscript.

## References

[B1] Kutz FW Frederick W Dobson RC Effects of temperature on the development of *Dirofilaria immitis* (Leidy) in *Anopheles quadrimaculatus* say and on vector mortality resulting from this development Ann Entomol Soc Am 1974 67 325 331

[B2] Cancrini G Pietrobelli M Frangipane di Regalbono A Tampieri MP della Torre A Development of *Dirofilaria* and *Setaria* nematodes in *Aedes albopictus* Parassitologia 1995 37 141 145 8778656

[B3] Cancrini G Gabrielli S Genchi C, Rinaldi L, Cringoli G Vectors of *Dirofilaria* nematods: biology behaviour and host/parasites relationships Proceeding of the First European Dirofilaria days: 22–25 February 2007; Zagreb, 2007 2007 Napoli: Rolando Editore 49 58

[B4] Morchón R Carretón E González-Miguel J Mellado-Hernández I Heartworm disease (*Dirofilaria immitis*) and their vectors in Europe - New distribution trends Front Physiol 2012 3 196 22701433 10.3389/fphys.2012.00196 PMC3372948

[B5] Genchi C Kramer LH Rivasi F Dirofilarial infections in Europe Vector Borne Zoonotic Dis 2011 11 1307 1317 10.1089/vbz.2010.0247 21417922

[B6] Otranto D Dantas-Torres F Brianti E Traversa D Petrić D Genchi C Capelli G Vector-borne helminths of dogs and humans in Europe Parasit Vectors 2013 6 16 10.1186/1756-3305-6-16 23324440 PMC3564894

[B7] Rossi L Pollono F Meneguz PG Cancrini G Four species of mosquito as possible vectors for *Dirofilaria immitis* piedmont rice-fields Parassitologia 1999 41 537 542 10870556

[B8] Muñnoz J Eritja R Alcaide M Montalvo T Soriguer RC Figuerola J Host-feeding patterns of native *Culex pipiens* and invasive *Aedes albopictus* mosquitoes (Diptera: Culicidae) in urban zones from Barcelona, Spain J Med Entomol 2011 48 956 960 10.1603/ME11016 21845962

[B9] Cancrini G Frangipane di Regalbono A Ricci I Tessarin C Gabrielli S Pietrobelli M *Aedes albopictus* is a natural vector of *Dirofilaria immitis* in Italy Vet Parasitol 2003 118 195 202 10.1016/j.vetpar.2003.10.011 14729167

[B10] Otranto D Capelli G Genchi C Changing distribution patterns of canine vector borne diseases in Italy: leishmaniosis vs. dirofilariosis Parasit Vectors 2009 2 Suppl 1 S2 10.1186/1756-3305-2-S1-S2 PMC2679394 19426441

[B11] Latrofa MS Montarsi F Ciocchetta S Annoscia G Dantas-Torres F Ravagnan S Capelli G Otranto D Molecular xenomonitoring of *Dirofilaria immitis* and *Dirofilaria repens* in mosquitoes from north-eastern Italy by real-time PCR coupled with melting curve analysis Parasit Vectors 2012 5 76 10.1186/1756-3305-5-76 22520170 PMC3438040

[B12] Service MW Mosquito ecology: field sampling methods 1993 2 London and New York: Elsevier Applied Science

[B13] Cancrini G Magi M Gabrielli S Arispici M Tolari F Dell’Omodarme M Prati MC Natural vectors of dirofilariasis in rural and urban areas of the Tuscan region, central Italy J Med Entomol 2006 43 574 579 10.1603/0022-2585(2006)43[574:NVODIR]2.0.CO;2 16739418

[B14] Mattingly PF Carthy JD, Sutcliffe JF The biology of mosquito-borne disease The Science of Biology (Series No I) 1969 London, United Kingdom: George Allen and Unwin Ltd

[B15] Coluzzi M Petrarca V Aspirator with paper cup for collecting mosquitoes and other insects Mosq News 1973 33 249 250

[B16] Snow KR Towards a checklist of British mosquitoes Entomol Mon Mag 1987 123 83 89

[B17] Gerberg EJ Ward RA Barnard DR Manual for Mosquito Rearing and Experimental Techniques. Bulletin No. 5 (revised) 1994 Lake Charles, Louisiana: Published by American Mosquito Control Association (AMCA), Inc 1 98

[B18] Favia G Dimopoulos G Della Torre A Touré YT Coluzzi M Louis C Polymorphism detected by random PCR distinguish between chromosomal forms of *Anopheles gambiae* Proc Natl Acad Sci 1994 91 22 10315 10319 10.1073/pnas.91.22.10315 7937947 PMC45010

[B19] Favia G Lanfrancotti A della Torre A Cancrini G Coluzzi M Polymerase chain reaction-identification of *Dirofilaria repens* and *Dirofilaria immitis* Parasitology 1996 6 567 571 8939053 10.1017/s0031182000067615

[B20] Preacher KJ Calculation for the chi-square test: An interactive calculation tool for chi-square tests of goodness of fit and independencehttp://quantpsy.org

[B21] Cowling DW Gardner IA Johnson WO Comparison of methods for estimation of individual-level prevalence based on pooled samples Prev Vet Med 1999 39 211 225 10.1016/S0167-5877(98)00131-7 10327439

[B22] EpiTools epidemiological calculators Available at: http://epitools.ausvet.com.au/content.php?page=home

[B23] Capelli G Poglayen G Bertotti F Giupponi S Martini M The host-parasite relationship in canine heartworm infection in a hyperendemic area of Italy Vet Res Commun 1996 20 320 330 10.1007/BF00366538 8865574

[B24] Lai CH Tung KC Ooi HK Wang JS Competence of *Aedes albopictus* and *Culex quinquefasciatus* as vector of *Dirofilaria immitis* after blood meal with different microfilarial density Vet Parasitol 2000 90 231 237 10.1016/S0304-4017(00)00242-9 10842003

[B25] Mulatti P Bonfanti L Capelli G Capello K Lorenzetto M Terregino C Monaco F Ferri G Marangon S West Nile Virus in North-Eastern Italy, 2011: Entomological and Equine IgM-Based Surveillance to Detect Active Virus Circulation Zoonoses Public Health 2011 Sep 13 10.1111/zph.12013 22971022

[B26] European Center for Disease Control (ECDC), VIBORNET mapsavailable at: http://ecdc.europa.eu/en/activities/diseaseprogrammes/emerging_and_vector_borne_diseases/Pages/VBORNET_maps.aspx

[B27] Cancrini G Romi R Gabrielli S Toma L DI Paolo M Scaramozzino P First finding of *Dirofilaria repens* in a natural population of *Aedes albopictus* Med Vet Entomol 2003 17 448 451 10.1111/j.1365-2915.2003.00463.x 14651660

[B28] Rebollar-Téllez EA Human body odor, mosquito bites and the risk of disease transmission Folia Entomol Mex 2005 2 247 265

[B29] Lacroix R Mukabana WR Gouagna LC Koella JC Malaria infection increases attractiveness of humans to mosquitoes PLoS Biol 2005 3 e298 10.1371/journal.pbio.0030298 16076240 PMC1182690

[B30] Cornet S Nicot A Rivero A Gandon S Malaria infection increases bird attractiveness to uninfected mosquitoes Ecol Lett 2012 Dec 3 10.1111/ele.12041 23205903

